# Different Members of the IL-1 Family Come Out in Different Ways: DAMPs vs. Cytokines?

**DOI:** 10.3389/fimmu.2013.00123

**Published:** 2013-05-24

**Authors:** Sonia Carta, Rosa Lavieri, Anna Rubartelli

**Affiliations:** ^1^Cell Biology Unit, IRCSS Azienda Ospedale Università San Martino-IST, Genoa, Italy

**Keywords:** IL-1α, IL-1β, IL-18, IL-33, secretion, damage associated molecular pattern, TLR, IL-1F receptors

## Abstract

Intercellular communications control fundamental biological processes required for the survival of multicellular organisms. Secretory proteins are among the most important messengers in this network of information. Proteins destined to the extracellular environment contain a signal sequence with the necessary information to target them to the Endoplasmic Reticulum, and are released by a “classical” pathway of secretion. However, in the early 1990s it became evident that non-classical mechanisms must exist for the secretion of some proteins, which in spite of their extracellular localization and function, lack a signal peptide. Indeed, the group of leaderless secretory proteins rapidly grew and is still growing. Many of them are implicated in the regulation of the inflammatory response. Interestingly, most members of the IL-1 family (IL-1F), including the master pro-inflammatory cytokine IL-1β, are leaderless proteins and find their way out of the cells in different manners. In this article, we will review current hypotheses on the mechanisms of externalization of IL-1F members and discuss their relevance with respect to the different functions (as cytokines or as DAMPs) played by the different IL-1 proteins.

## The IL-1 Family

IL-1 family (IL-1F) is evolutionary ancient. Eleven IL-1 members have been identified (Table 1) based on conservation of amino acid sequence, identity of gene structure, and three-dimensional structure (Dunn et al., 2001). Most of them (with the exception of IL-18 and IL-33, Nolan et al., 1998; Schmitz et al., 2005) map to chromosome 2 between the IL-1α and IL-1 Receptor antagonist (IL-1ra) loci (Nicklin et al., 2002), suggesting that each IL-1F member derives from the duplication of a common ancestral gene. Each IL-1 gene codes for a protein that contains a single structural domain formed from 12 beta strands connected by loop regions arranged in a beta-trefoil structure. IL-1F members differ most from each other within these loop regions (Dunn et al., 2001).

**Table 1:** **IL-1 family members**.

Family name	Name	Receptor/coreceptor	Property	Synthesized as precursor	Processing required for activity
IL-1F1	IL-1α	IL-1RI/IL-1RacP	Proinflammatory	Yes	No
IL-1F2	IL-1β	IL-1RI/IL-1RacP	Proinflammatory	Yes	Yes
IL-1F3	IL-1Ra	IL-1RI	Antagonist for IL-1α,β	No	No
IL-1F4	IL-18	IL-18Rα/IL-18Rβ	Proinflammatory	Yes	Yes
IL-1F5	IL-36Ra	IL-1Rrp2	Antagonist for IL-36	Yes	Yes
IL-1F6	IL-36α	IL-1Rrp2/IL-1RAcP	Proinflammatory	Yes	Yes
IL-1F7	IL-37	IL-18Rα, IL18BP	Anti-inflammatory	Yes	Yes
IL-1F8	IL-36β	IL-1Rrp2/IL-1RAcP	Proinflammatory	Yes	Yes
IL1-F9	IL-36γ	IL-1Rrp2/IL-1RAcP	Proinflammatory	Yes	Yes
IL-1F10	IL-38	IL-1Rrp2	? Antagonist	Yes	? No
IL-1F11	IL-33	ST2/IL-1RAcP	Proinflammatory	Yes	No

The various members of the IL-1F play different biologic activities all involved in innate immunity (Dinarello, 2009). Interestingly, although most IL-1F proteins are proinflammatory, also members endowed with anti-inflammatory properties exist, the most important being IL-1ra (Arend et al., 1998).

Most IL-1 family members share features which make them different from the other cytokines. First of all, they are synthesized as precursor proteins that subsequently undergo proteolytic maturation by converting enzymes. Proteolytic maturation is strictly required for activation of some members of IL-1 family, such as IL-1β, IL-18 (Dinarello, 1998a), and IL-37 (Boraschi et al., 2011). In the case of other IL-1F members, the precursor is able to engage its receptor and trigger a response on target cells. This is the case of IL-1α (Dinarello, 1998b) and IL-33 (Moussion et al., 2008). The major converting enzyme responsible for processing of IL-1β, IL-18, and IL-37 is caspase-1 (Black et al., 1988; Ghayur et al., 1997; Kumar et al., 2002). This convertase is produced as a zymogen (pro-caspase-1) and undergoes activation upon the assembly of intracellular multiprotein complexes named inflammasomes (Bauernfeind et al., 2011). Different types of Inflammasomes exist, each composed by a member of the nucleotide-binding domain leucine-rich repeat containing (NLR) gene family, adaptor proteins, and pro-caspase-1 molecules.

A second, important feature of IL-1F proteins is that only IL-1ra is a classical secretory protein endowed with a signal peptide: all the other members are leaderless (Dinarello, 2009).

## Leaderless IL-1 Cytokines: How Do They Get Out of Cells?

In principle, leaderless proteins, synthesized in the soluble cytosol, should stay there: a quite unlikely behavior for soluble mediators of inflammation. Alternatively, they must find a way out different from the classical secretory pathway. When the gene of IL-1β was cloned revealing the absence of a signal sequence (Auron et al., 1984) the first explanation for IL-1β externalization was that it was simply released by cells dying at the site of inflammation. This hypothesis was ruled out many years ago by two major evidences (Muesch et al., 1990; Rubartelli et al., 1990): (i) IL-1β is selectively released by LPS activated human monocytes: only the mature form of IL-1β, but neither the IL-1β precursor (pro-IL-1β) nor other cytosolic proteins are detectable in culture supernatants; (ii) viable cells are required for secretion of mature IL-1β: when activated monocytes were killed by freezing and thawing before incubating at 37°C, only pro-IL-1β accumulated in the culture supernatant.

Further studies confirmed that an active secretory pathway, different from the ER-Golgi one, exists for IL-1β and also for IL-18 (Andrei et al., 1999; MacKenzie et al., 2001; Qu et al., 2007). Death as a mechanism of secretion was instead proposed for other members of the IL-1F, such as IL-1α and confirmed by various studies Sakurai et al. (2008), Luheshi et al. (2009), and Cohen et al. (2010).

## Secretion of IL-1β and IL-18

**Figure 1:**
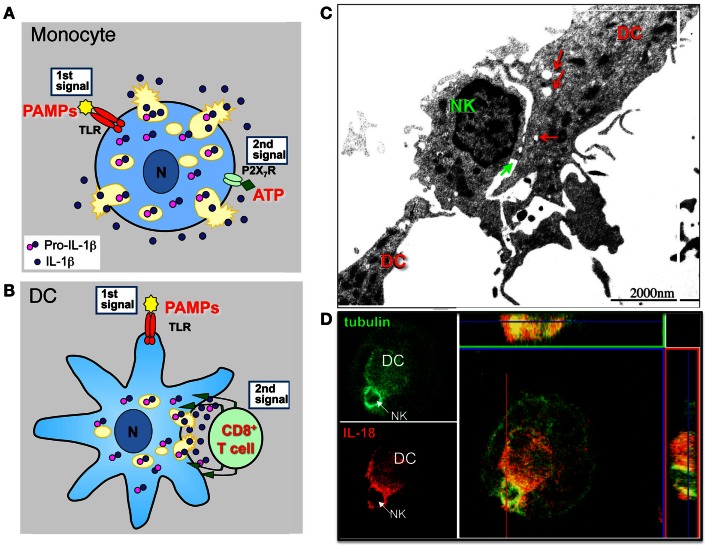
**Lysosome exocytosis allows diffuse and polarized secretion of IL-1β and IL-18**. **(A)** Models of non-polarized secretion of IL-1β. TLR agonists (e.g., PAMPs) induce monocytes/macrophages to actively synthesize pro-IL-1β that accumulates into the cytosol and in part into secretory lysosomes. A second extracellular soluble signal (e.g., ATP) triggers generalized lysosome exocytosis (Andrei et al., 1999). A similar mechanism accounts for IL-18 secretion (Perregaux et al., 2000). **(B)** Models of polarized (lower panel) secretion of IL-1β. In DCs, a first maturative stimulus (e.g., TLR triggering), induces pro-IL-1β synthesis. The second signal is provided by antigen specific T cells that induces a [Ca^2+^]_i_ rise, followed by recruitment of IL-1β-containing secretory lysosomes toward the interacting T cell, and by exocytosis restricted to the intercellular space (immunological synapse) (Gardella et al., 2000b, 2001). A similar mechanism mediates IL-18 secretion in DCs interacting with autologous NK cells (Semino et al., 2005). **(C)**. Electron microscopy analysis of a DC interacting with a NK cell. Interaction between the two cells occurs primarily in correspondence with DC areas enriched by mitochondria and vesicles (red arrows). The immunological synapse is indicated by the green arrow. **(D)** Confocal microscopy analysis of tubulin (green) and IL-18 (red) in a DC/NK conjugate after 3 h of interaction. The strong co-staining of IL-18 and tubulin in both transversal and sagittal sections indicates that IL-18 from DC polarizes toward the NK/DC synapse and is transported along tubulin filaments. **(C,D)**: modified from Semino and Rubartelli (2010).

Secretion of IL-1β and IL-18 needs two signals (Figure 1A). A first signal, supplied by bacterial products that bind and activate Toll-like Receptors (TLR), triggers IL-1β expression and synthesis; this signal is needed also for IL-18 secretion even though IL-18 is constitutively expressed by myeloid cells (Perregaux et al., 2000). The second signal is provided by a variety of diverse stimuli: endogenous, such as extracellular ATP, or exogenous, such as microbial products or pathogenic crystals (Bauernfeind et al., 2011). However, while in murine macrophages a second signal is strictly required, in primary human monocytes the addition of second signals such as ATP strongly enhances secretion but is dispensable as bacterial products alone are sufficient to induce secretion of IL-1β, although at a lower extent and with slow kinetics (Piccini et al., 2008). In fact upon TLR-triggering, human monocytes externalize functionally effective amounts of their ATP that in turn stimulates autocrinally the monocyte purinergic P2 × 7 receptor, triggering the cascade of events that lead to inflammasome activation and IL-1β secretion (Piccini et al., 2008).

Several models of IL-1β and IL-18 secretion have been proposed but the precise mechanism remains elusive. In particular, where and how inflammasome activation and processing of the two pro-cytokines occur, as well as the link between processing and secretion are still unknown (Rubartelli, 2012). Most of the described IL-1β secretory pathways involve the externalization of the cytokine via vesicles (Andrei et al., 1999; MacKenzie et al., 2001; Qu et al., 2007). Secretory lysosomes, microvesicles shed from the plasma membrane, and exosomes have been identified as vesicles able to carry IL-1β out of the cell in different cell types (primary monocytes, monocyte continuous cell lines, mouse macrophages).

An additional, non-vesicular pathway of IL-1β secretion may take place in monocyte/macrophages where sustained activation of the NLRP3 or NLRC4 inflammasome cascade induces caspase-1-mediated pyroptotic death (Le Feuvre et al., 2002; Brough and Rothwell, 2007; Bergsbaken et al., 2009). In these cells, a direct efflux of cytosolic mature IL-1β occurs across hyper-permeable plasma membranes.

It is possible that differences in the cell type, in the functional state of the cells or in the culture conditions, as well as strength and duration of the stimulus (Carta et al., 2011; Lopez-Castejon and Brough, 2011) may account for the different vesicular or non-vesicular IL-1β secretory pathways used.

The first suggested mechanism is the secretory lysosome-dependent pathway, characterized in our lab on primary human monocytes (Andrei et al., 1999, 2004). In this model, pro-IL-1β is translocated together with caspase-1 into vesicles belonging to the endolysosomal compartment. The mature form of IL-1β is produced within the vesicles by caspase-1 cleavage, after which the endolysosomes fuse with the plasma membrane and the content is released into the extracellular space. The capacity to fuse with plasma membrane and to externalize the soluble content is a peculiarity of a subset of endolysosomes, called secretory lysosomes (Blott and Griffiths, 2002). These are Ca^2+^-regulated secretory organelles displaying features of both classical endolysosomes and secretory granules responsible for regulated secretion in specialized cells (Blott and Griffiths, 2002). Particularly abundant in hemopoietic cells they participate in inflammatory and immune response by mobilizing their content into the external milieu in response to triggering signals. For instance, CTL and NK cells destroy their infected or tumor target cells by secreting cytolytic proteins, which are stored in secretory lysosomes.

Other leaderless proteins may be imported into cytoplasmic organelles related to the lysosomal compartment in myelomonocytic cells. Among IL-1F members, IL-18 seems to follow the same route as IL-1β (Semino et al., 2005). Also HMGB1, another inflammatory mediator, is present into endolysosomal related organelles of activated monocytes (Gardella et al., 2002). Given the implication of secretory lysosomes in many immune-inflammatory processes (Blott and Griffiths, 2002), lysosome-mediated secretion of IL-1β, IL-18, HMGB1 is consistent with the role played by these proteins in the modulation of innate immunity. Interestingly, the involvement of acidic vesicles in the export of leaderless proteins is evolutionary conserved as in *Dictyostelium discoideum*, translocation into exocytic contractile vacuoles of DdCAD-1, a leaderless adhesion protein, is necessary for its externalization (Sesaki et al., 1997).

## Lysosome-Mediated Polarized Secretion

In general, lysosome-mediated secretion is a regulated process in that a triggering signal is required to induce exocytosis (Blott and Griffiths, 2002). In the case of IL-1β, we have shown that LPS induce synthesis of pro-IL-1β with cytosolic accumulation and lysosomal translocation, then exogenous ATP triggers with IL-1β release (Andrei et al., 2004). A similar two step mechanism seems to account for the regulated secretion of IL-18 (Perregaux et al., 2000) and HMGB1 (Gardella et al., 2002). In all these cases, the signal triggering secretion is generated during the process of inflammation: ATP, promoting IL-1β and IL-18 secretion (Laliberte et al., 1997; Perregaux et al., 2000), is released by monocytes themselves after TLR stimulation and by other cells involved in inflammation (i.e., platelets) (Ferrari et al., 1997; Piccini et al., 2008); differently, active phospholipids such as phosphatidylcholine, possible responsible for secretion of HMGB1, appear later in the inflammatory microenvironment (Gardella et al., 2002).

Interestingly, not only inflammatory cells such as monocytes but also mature dendritic cells (DCs), the professional antigen presenting cells, express inflammatory leaderless cytokines such as IL-1β and IL-18. In these cells, secretion may be induced by antigen specific T cells (Gardella et al., 1999, 2000a,b, 2001) or NK cells (Semino et al., 2005). Morphological approaches allowed to demonstrated that interaction between DCs and CD8^+^ T cells (Gardella et al., 2001) or NK cells (Semino et al., 2005) is associated with recruitment of IL-1β or IL-18-containing secretory lysosomes in the area of contact among the cells followed by polarization of these organelles, with evidence of lysosome exocytosis at the intercellular space, the so called “immunological synapse” (Figures 1B–D). These findings deserve two considerations. On the one hand, they underline the existence of a bidirectional cross talk between DCs and T lymphocytes or NK cell specifically interacting with them, in which the T or the NK cell induce the functional polarization of the DC and the DC responds by degranulation oriented toward the same interacting T or NK cell, with obvious relevance for the control of the immune response. On the other hand, the different way of regulating secretion by monocytes and DCs may account for the different function of IL-1β and IL-18 in inflammation and immune response (Figure 1). Monocytes respond to soluble signals with generalized exocytosis, thus allowing the spreading of inflammatory cytokines in the microenvironment (Figure 1A). DCs respond to the localized signal provided by the interacting T or NK cell (Figures 1B–D). This restricts the area of release to the immunological synapse and allows activation of target cells without spreading of the cytokine, thus controlling inflammation. Thus, lysosome-mediated secretion of inflammatory leaderless proteins allows polarized secretion in non-polarized cells (Chimini and Rubartelli, 2005).

## Autophagy and IL-1β and IL-18 Secretion: Lysosome Exocytosis Revisited?

**Figure 2:**
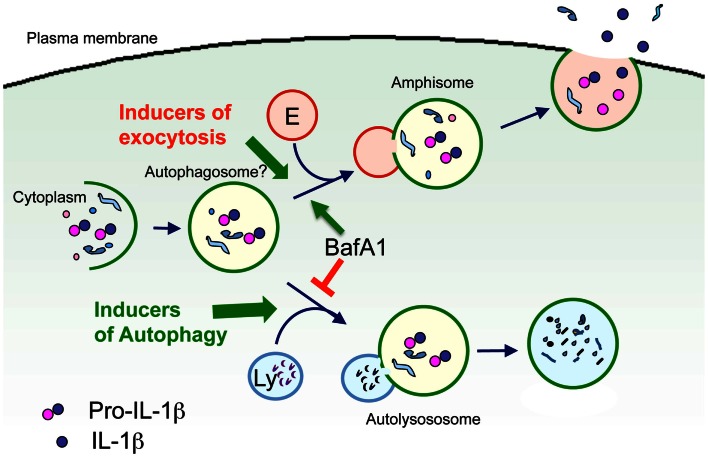
**Model of autophagosome involvement in IL-1β secretion**. Cytosolic pro-IL-1β is sequestered in part into vesicles belonging to the autophagosomal-lysosomal compartment (autophagosomes?). Autophagosomes may then fuse either with lysosomes (Ly), resulting in degradation of pro-IL-1β, or with endosomes (E) to form amphisomes, were pro-IL-1β may undergo processing. Amphisomes then fuse with the plasma membrane leading to exocytosis of IL-1β. Type and strength of the stimuli may determine the fusion with either endosomes or lysosomes, and thus dictate the fate of pro-IL-1β toward degradation or secretion. Support to this hypothesis comes from the observation that the vacuolar H+ ATPase inhibitor bafilomycin A1 (BafA1) that prevents the fusion between autophagosomes and lysosomes, promotes IL-1β secretion (Sonia Carta and Anna Rubartelli, unpublished results).

Autophagy preserves the correct quality and quantity of the eukaryotic cytoplasm through two main highly conserved mechanisms: (i) cytosol autodigestion during starvation, which ensures cell-autonomous provision of energy and nutrients; (ii) removal of old organelles and aggregates exceeding the capacity of other cellular degradative systems (Levine and Kroemer, 2008). Recently, an involvement of autophagy in the process of leaderless secretion has been proposed. In fact, the secretion of the yeast leaderless secretory protein Acb1 was strongly enhanced by treatments that induce autophagy (nitrogen starvation or rapamycin). Accordingly, strains mutant for different key factors of autophagy are deficient in Acb1 secretion (Duran et al., 2010; Manjithaya et al., 2010). These results suggest that Acb1 is sequestered in autophagosomes that do not fuse with the vacuole but with endosomes to form amphisomes. Amphisomes might, in turn, fuse with the plasma membrane leading to Acb1 release in the extracellular medium (Giuliani et al., 2011). In agreement with the data on yeast, a link between autophagy and secretion of IL-1β is being emerging. In principle, the “secretory lysosomes” that were formerly found to containing IL-1β and caspase-1 may well represent autophagosomes, and like autophagosomes, may be destined either to fusion with lysosomes, and thereby to autophagic degradation of their cargoes, or to fusion with the plasma membrane, with externalization of their cargoes (Figure 2).

However, how autophagy regulates IL-1β secretion is highly debated. Both articles suggesting that autophagy inhibits secretion (Saitoh et al., 2008; Crisan et al., 2011; Harris et al., 2011; Zhou et al., 2011; Shi et al., 2012) and viceversa, that autophagy is required for IL-1β secretion (Dupont et al., 2011) have been published.

The hypothesis of autophagy as a positive mediator of IL-1β secretion is more appealing, according to the following considerations: (i) TLR triggering, a crucial step in IL-1β secretion, which is both necessary and sufficient to drive IL-1β synthesis, maturation, and secretion in human monocytes (Piccini et al., 2008), induces autophagy in myelomonocytic cells (Xu et al., 2007; Delgado et al., 2008); (ii) the hypothesis is consistent with the vesicular models of IL-1β secretion (Andrei et al., 1999; Qu et al., 2007). Besides, autophagy as a blocking mechanism for IL-1β secretion is supported by clear *in vitro* data, such as the strong inhibition of IL-1β secretion induced by substances that promote autophagy and, conversely, the enhancement observed with compounds that block autophagy (Crisan et al., 2011; Harris et al., 2011; Shi et al., 2012) and by the results obtained in mice deficient of autophagy genes (Saitoh et al., 2008; Nakahira et al., 2011).

A possible explanation for these contrasting findings is that formation of pro-IL-1β containing autophagosomes is a pre-requisite for IL-1β secretion, and it is induced by TLR activation. Autophagosomes may then undergo exocytosis, with secretion of IL-1β, or fuse to lysosomes, with degradation of pro-IL-1β, depending on the type and strength of the stimuli that trigger IL-1β producing cells. Thus, strong stimuli (e.g., high doses of TLR agonists) or exposure to inducer of autophagy such as rapamycin, would privilege maturation of autophagosome to autolysosome, and degradation of its content by the hydrolases provided by lysosomes. Weaker stimuli, such as continuous triggering by lower doses of TLR agonists would instead favor amphisome fusion with the plasma membrane, resulting in secretion (Figure 2).

## The Travel of Cytokines from Inside to Outside during Evolution. Both Passive Release and Active Secretion Account for IL-1α and IL-33 Externalization

The founding member of IL-1 family is probably IL-1α because of its close homology to acidic FGF, in turn one of the most ancient cytokines. It is conceivable that, at the beginning, IL-1F cytokines were, like FGF, intracellular growth factors, and repair molecules interacting with DNA as transcription factors (Dinarello, 2010) and were passively externalized by dying cells. When later in evolution Immunoglobulins appeared, they were used by extracellular cytokines/growth factors as cell surface receptors, and cytokine-mediated signaling evolved. Some IL-1F members including IL-1α and IL-33 retain intracellular (nuclear) function (Carriere et al., 2007; Cayrol and Girard, 2009). Interestingly, unlike IL-1β and IL-18, IL-1α, and IL-33 can activate their receptors on target cells as full-length molecules: thus when released from injured cells they can exert their biological activity in the absence of a proteolytic processing (Chen et al., 2007; Eigenbrod et al., 2008; Cayrol and Girard, 2009).

Based on the above observations, IL-1 family members can be divided into a group of cytokines that retain some intracellular function and are passively externalized upon cell lysis (the prototype being IL-1α), and a second group including cytokines that are stored into the cell cytosol before secretion, but do not play intracellular function and undergo regulated processing and secretion (the prototype being IL-1β).

Still, the situation is more complex. In fact, a number of reports indicate the possibility that also IL-1α and IL-33 are actively released by cells that maintain their integrity. IL-1α was reported to be secreted in response to heat shock (Mandinova et al., 2003) and through an unknown mechanism requiring caspase-1 (Gross et al., 2012). In the case of IL-33, intracellular calcium increase, regulated autocrinally by ATP and purinergic receptor stimulation, induces translocation from nucleus to cytoplasm and release of full-length IL-33 (Kouzaki et al., 2011). Extracellular ATP is a well-known inducer of inflammasome activation and IL-1β/IL-18 processing. The mechanism through which ATP induces IL-33 secretion seems to be different, since the unprocessed, full-length molecular form of IL-33 is secreted. However, we have previously observed that in human monocytes ATP drives exocytosis of pro-IL-1β containing vesicles also if caspase-1 is inhibited, resulting in secretion of the precursor form of the cytokine (Andrei et al., 2004). Moreover, both in monocytes and in DCs, calcium influx induces secretion of pro-IL-1β and pro-IL-18 (Gardella et al., 2000a, 2001; Andrei et al., 2004). Thus it is conceivable that the pathway described for IL-33 makes use of mechanisms (purinergic receptor stimulation and calcium influx) which are old and conserved during evolution. The ATP-mediated signaling may then have further specialized adding to the older function of inducing exocytosis the newer function of controlling inflammasome activation and hence bioactivity of cytokines such as IL-1β and IL-18.

Although the study by Kouzaki et al. (2011) did not investigate the subcellular localization of IL-33 after its nucleus-cytoplasmic translocation in airway epithelial cells, a recent report (Kakkar et al., 2012) indicates that, in fibroblasts, newly synthesized IL-33 first moves to the nucleus and then is translocated to cytoplasmic vesicles, a pathway reminiscent of that followed by HMGB1 (Gardella et al., 2002). Secretion of uncleaved IL-33 is induced by mechanical strain (i.e., application of a physical deformation) in the absence of cellular necrosis (Kakkar et al., 2012). Extracellular release of IL-33 is also observed in mice subjected to acute transaortic constriction, which causes mechanical stress in the left ventricle (Kakkar et al., 2012). Together, these data suggest that IL-1α and IL-33 may be secreted by cells that are subjected to non-lethal stress in addition of being released by necrotic cells.

## Evolution of Leaderless Secretion

Mechanisms of secretion of leaderless proteins were probably exploited by cells to get rid of proteins which can be harmful, either because they are misfolded, or too abundant, or mislocated. Such mechanisms exists in yeast, where toxic proteins are removed from the cytoplasm through a non-classical mechanism of secretion (Cleves et al., 1996). A similar mechanism operates also in mammals: for instance, the sulfotransferase rhodanese, which in physiological conditions accumulates in mitochondria, when overexpressed is rapidly externalized by transfected cells without any sign of cell lysis (Sloan et al., 1994). Similarly, expression of Green Fluorescent Protein (GFP) results in cytosolic accumulation of properly folded protein but also activates the secretion of misfolded GFP molecules (Tanudji et al., 2002). Leaderless secretion may thus act as a safety valve, maintaining cellular homeostasis when the cytoplasmic degradative pathways are overloaded.

## Stress as a Common Inducer of IL-1F Member Leaderless Secretion

As discussed above the various members of the IL-1 family have exploited many different ways to get out of cells. However, all of them seem to be switched on by cellular stress due to changes in environmental conditions (Giuliani et al., 2011). This is true both for the passive release by dying cells exploited by IL-1α and IL-33, since death represents the last step of a cell subjected to stress, and for the more complex secretion of IL-1β and IL-18 that are regulated by redox stress (Cruz et al., 2007; Dostert et al., 2008; Hewinson et al., 2008; Meissner et al., 2008; Tassi et al., 2009). Interestingly, both purinergic receptor stimulation and mechanical stress, cause a disturbance of redox homeostasis resulting in redox response (Wu et al., 2013); redox response is also required for inflammasome activation (Rubartelli, 2012). Interestingly, a recent study shows that mechanical stress, which as stated above induces IL-33 secretion, is sensed by NLRP3 inflammasome and leads to IL-1β processing and secretion (Wu et al., 2013). Mechanical stretch induces production of Reactive Oxygen Species (ROS) which are well-known players in the mechanism of NLRP3 inflammasome activation (Rubartelli, 2012). ROS production is also a very ancient cell defense mechanism (Naviaux, 2012) and induce redox signaling (Carta et al., 2009). It is possible that, depending on the cell type, the redox signaling is different, resulting in different effects on cytokine processing/release. For instance, while in professional inflammatory cells the evolution of the inflammasome complex favors redox-mediated processing of IL-1β and IL-18, in airway epithelial cells or fibroblasts the redox response could only induce cytokine externalization.

Stress as an inducer of secretion of many IL-1 family members is in agreement with the fact that the interleukin (IL)-1 family more than any other cytokine family is closely linked to the innate immune response, that is, to the first line of host defense against stressful noxia (Dinarello, 2009). This link became evident upon the discovery that the cytoplasmic domain of the IL-1 receptor type I is highly homologous to the cytoplasmic domains of all TLRs (Medzhitov et al., 1997). Fundamental inflammatory responses such as the induction of cyclooxygenase type 2, increased expression of adhesion molecules, or synthesis of nitric oxide are indistinguishable responses of both IL-1 and TLR ligands (Dinarello, 2009). Thus, IL-1F members are a “frontline” emergency cytokines produced very early in response to multiple stresses.

## IL-1F Members: Borderline Between DAMPs and Cytokines

Another group of early inducers of inflammation are DAMPs. These are usually nuclear or cytosolic proteins, with a defined intracellular function that, when released by stressed cells undergoing necrosis, act as endogenous danger signals and initiate and perpetuate inflammation (Lotze et al., 2007). This is possible because DAMPs binds to specific receptors, unrelated to their intracellular function, whose engagement triggers inflammatory responses on target cells. The DAMPs features listed above are very similar to those associated to some IL-1F members, particularly IL-1α and IL-33. In fact, unlike IL-1β which is induced in a restricted number of inflammatory cells by inflammatory stimuli and undergoes regulated secretion, IL-1α and IL-33 are constitutively expressed and accumulate in large amounts in “barrier tissues” (epithelial and endothelial), the first ones entering in contact with external noxia (Dinarello, 2009; Liew et al., 2010). Both IL-1α and IL-33, although constitutively expressed, are further induced by stress conditions, thus increasing the amount of inflammatory mediator ready to be released in case of need. Interestingly, also certain DAMPs, such as HMGB1 are further increased in cells exposed to stress (Lotze et al., 2007).

The difference between cytokines such as IL-1α and IL-33 and DAMPs is therefore a moot point. The argument that DAMPs are only released by dying cells, whereas IL-1α and IL-33 can also be actively secreted has been discarded by the demonstration that the DAMP HMGB1 undergoes regulated secretion by certain cell types (Gardella et al., 2002). Actually, more recent data indicate that secretion of HMGB1, as well as of other leaderless secretory proteins not belonging to IL-1F, is also dependent on caspase-1 and inflammasome (Keller et al., 2008; Willingham et al., 2009; Lamkanfi et al., 2010). However, the underlying mechanism is unknown.

Rather, an important difference between DAMPs and IL-1F cytokines are the receptors. DAMPs bind to Pattern Recognition Receptors (PRR) such as TLRs (Leadbetter et al., 2002; Park et al., 2006; Yu et al., 2006), the most ancient membrane bound, and intracellular receptors that detect microbial invasion and initiate innate immune defenses (Kawai and Akira, 2010). These receptors are promiscuous because of their tendency to associate with different domains (Kawai and Akira, 2010), including domains present in DAMPs. During evolution, the appearance of PRR and their capacity to bind and be activated by some proteins released by dying cells, have provided a second life to these proteins, which became DAMPs.

IL-1F members have specific receptors belonging to the Toll-IL-1 receptor (TIR) superfamily by virtue of their intracellular signaling domain, shared with TLRs (O’Neill, 2008). These receptors are endowed with three extracellular immunoglobulin (Ig)-like repeats that bind IL-1F cytokines with high affinity. This implies that, at variance with DAMPs, a relatively low concentration of a given IL-1F cytokine is adequate to trigger a physiological response. The presence of specific receptor antagonists (i.e., IL-1ra) and binding proteins (i.e., IL-18 binding protein), which prevent cytokine-receptor interaction ensures a better control of the inflammatory response than in the case of DAMPs (Dinarello, 2009).

IL-1F receptors evolved after PRR. Before, cytokines worked as intracellular growth and repair molecules. Thus, in organisms (such as starfish) expressing IL-1 like molecules (Beck and Habicht, 1986) and TLRs but not cytokine receptors, an IL-1 like molecule, externalized by injured or stressed cells, was just diluted in the water and lost. Only when Ig appeared and cytokine receptors evolved, IL-1F members became able to work extracellularly as soluble mediators.

The independent evolution of leaderless secretion aimed at eliminating harmful intracellular proteins while maintaining cell integrity and of Ig superfamily members that specifically bind some leaderless secretory proteins, converged to assist in host defense: proteins fought off cells because no longer useful, became cytokines.

In conclusion, since TLR receptors are evolutionary more ancient than IL-1F receptors, it is conceivable that DAMPs are the first inflammatory mediators appeared along evolution. However, at variance with TLRs, IL-1F receptors are high affinity and non-promiscuous receptors. Although some IL-1F cytokines are passively released by dying cells, the production and activity of other IL-1F members (IL-18, IL-1β) is strictly regulated. Moreover, increasing evidence indicates that also IL-1α and IL-33, classically considered as passively released, can be actively secreted by stressed cells. Lack of promiscuity and control of cytokine activity at several levels allows the generation of a complex network of cytokines that ensures the correct development and the positive outcome of inflammatory responses. Thus, although DAMPs are indeed able to trigger and perpetrate inflammation, the evolution of cytokines provided a strong impulse to innate immunity.

## Conflict of Interest Statement

The authors declare that the research was conducted in the absence of any commercial or financial relationships that could be construed as a potential conflict of interest.
